# Antimicrobial resistance in urinary isolates from inpatients and outpatients at a tertiary care hospital in South-Kivu Province (Democratic Republic of Congo)

**DOI:** 10.1186/1756-0500-7-374

**Published:** 2014-06-18

**Authors:** Leonid M Irenge, Landry Kabego, Olivier Vandenberg, Raphael B Chirimwami, Jean-Luc Gala

**Affiliations:** 1 Bukavu General Hospital/Université Catholique de Bukavu, P.O. Box 285, Bukavu, Democratic Republic of Congo; 2 Center for Applied Molecular Technologies, Institut de Recherche Expérimentale et Clinique, Université catholique de Louvain, Clos chapelle-aux-champs, 30 B1.30.24, 1200 Brussels, Belgium; 3 Defense Laboratories Department, ACOS Ops&Trg, Belgian Armed Forces, Martelarenstraat, 181, 1800 Peutie, Belgium; 4 Infectious Diseases Epidemiological Unit, Public Health School, Université Libre de Bruxelles, Brussels, Belgium

**Keywords:** Urinary tract infections, South-Kivu, Democratic Republic of Congo, Multidrug resistance, Prevalence, Extended spectrum beta-lactamases, Antibotics

## Abstract

**Background:**

The rate of antimicrobial resistant isolates among pathogens causing urinary tract infections (UTIs) in Democratic Republic of Congo (DRC) is not known. The aim of the current study was to determine this rate at the Bukavu Provincial General Hospital (province of South-Kivu, DRC).

**Findings:**

A total of 643 isolates (both from inpatients and outpatients) collected from September 2012 to August 2013 were identified using biochemical methods, and tested for antimicrobial susceptibility. The isolates were further screened for Extended-Spectrum Beta-Lactamases (ESBL) production. Beta-lactamase AmpC phenotype was investigated in 20 antibiotic-resistant isolates.

*Escherichia coli* (58.5%), *Klebsiella spp. (*21.9%) and *Enterobacter spp.* (16.2%) were the most frequent uropathogens encountered. Rare uropathogens included *Citrobacter spp.*, *Proteus spp.,* and *Acinetobacter spp*. Resistance was significantly more present in inpatients isolates (22.1% of isolates) when compared to outpatients isolates (8.4% of isolates), (p-value <0.001). Antibiotic-resistant isolates displayed resistance to common antimicrobial drugs used for UTIs treatment in South Kivu province, namely: ciprofloxacin, ampicillin and third generation cephalosporins. ESBL-phenotype was present in 92.9% of antibiotic-resistant isolates. Only amikacin, nitrofurantoin and imipenem displayed satisfactory activity against antibiotic resistant isolates.

**Conclusions:**

This study confirms the presence of antibiotic-resistant uropathogens (mainly ESBL-producers isolates) at the Bukavu General Hospital. This study should serve as a wake-up call and help to raise awareness about the threat to public health of antibiotic resistance in this DRC province.

## Introduction

Urinary tract infections (UTIs) refer to any type of urothelial inflammatory response resulting from the invasion of the urinary tract by microbial pathogens [1,2]. They are considered to be the most common bacterial infections worldwide, whether in the community or hospital setting [3-5]. While UTIs are well documented worldwide [6-20], there are currently no reports on UTIs prevalence in DRC, the second largest Africa country. The country has been plagued by a protracted civilian war which has claimed an estimated five million lives [21], with nearly 60% of all deaths caused by infectious diseases [22-24].

Accordingly, the aim of this study was to monitor the rate of resistant urinary pathogens isolated from patients with UTIs (both from community and hospital settings) attending Bukavu Provincial Hospital (South Kivu, DRC) from September 2012 to August 2013, as well as to determine the pattern of antibiotic resistance to commonly used antimicrobial agents in the province.

## Materials and methods

### Study population and bacterial isolates

This cross-sectional study was conducted in both outpatients and inpatients suspected of UTI at the Bukavu Provincial Hospital. This hospital is one of the main healthcare facilities of Bukavu, a city of more than 500 000 inhabitants. The hospital also serves as the health reference center for the province of South-Kivu. It has 385 beds with 6400 admissions and 4900 outpatients per year.

Urinary samples were collected in sterile universal containers from patients presenting with UTI symptoms (dysuria, pelvic pain, with or without fever) from September 2012 to August 2013. Exclusion factors were structural or functional abnormalities of the genitourinary tract. Catheterized patients were also excluded. Samples were cultured within 30 minutes of collection. Samples displaying pyuria (white blood cell count greater than 5 per high-power field upon light microscopic examination) and/or bacteriuria 10^5^ CFU per mL were further processed for culture and subsequent antimicrobial susceptibility testing. Each specimen was cultured using a 10 μL calibrated loop to inoculate cysteine lactose electrolytes deficient (CLED) agar and incubated at 37°C for 16–24 hours and the number of colonies was counted. A growth of >10^5^ colony forming units/mL of one type of organism was considered as significant bacteriuria. Identification was performed using standard biochemical tests [25].

Ethical approval for the study was granted by the Institutional Review Board (IRB) of the Université Catholique de Bukavu, DRC. The study complied with the World Health Organization and international guidelines on antibiotic surveillance for which no recommendation for an informed consent has been issued. In order to ensure confidentiality, samples were analyzed anonymously.

### Antimicrobial susceptibility testing

The isolates were tested by the disk diffusion method on Muller Hinton agar II and the results were interpreted according to the guidelines European Committee on Antimicrobial Susceptibility Testing (EUCAST, 2013) [26]. Antibiotic disks were purchased from Bio-Rad (Nazareth Eke, Belgium). The following antibiotics were tested: amikacin, ampicillin, amoxicillin, amoxicillin-clavulanic acid, ceftriaxone, ceftazidime, imipenem, trimethoprim-sulfamethoxazole, amikacin, ciprofloxacin, nitrofurantoin. Isolates showing resistance to at least one cephalosporin were tested for ESBL production by the double-disk synergy test on Mueller-Hinton agar (Biorad, Nazareth Eke; Belgium) using ceftazidime and ceftriaxone placed at a distance of 20 mm apart from a disk containing amoxicillin plus clavulanic acid. A clear-cut enhancement of the inhibition in front of either ceftazidime and ceftriaxone disks towards the clavulanic acid-containing disk (also called “champagne-cork” or “keyhole”) was interpreted as positive for ESBL production [27]. E-test strips (BioMérieux, Marcy l’Etoile, France) were used for confirmation of ESBL production. Minimum inhibitory concentrations (MIC) of cefotaxime and ceftazidime with and without clavulanic acid were determined, after 16–18 hours incubation on Mueller Hinton plates inoculated with suspension of isolates at a fixed density (0.5 to 0.6 McFarland standard). The test was performed and interpreted according to the manufacturer’s instructions. *Escherichia coli* ATCC 35218 and *Klebsiella pneumoniae* ATCC 700603 strains were used as ESBL negative and positive controls respectively. Twenty ampicillin and/or third-generation cephalosporins-resistant isolates (n = 20) were tested for the presence of the beta-lactamase AmpC phenotype, using cefoxitin-cloxacillin disk diffusion test as described by Tan *et al.*[28]. Multi-drug resistance was defined as non-susceptibility to at least one agent in three or more antimicrobial categories [29]. All multi-drug resistant isolates were cryopreserved at -80°C for further studies.

### Statistical analysis

Statistical analyses were performed using the SPSS statistical package release 12.0 for Windows (SPSS, Inc., Chicago, IL). Differences in group proportions and categorical variables were assessed using the chi-square test. A *p*-value <0.05 was considered as statistically significant.

## Findings

Clean-catch midstream urine specimens (n = 2724) were processed during the 12-month study period (from September 1^st^ 2012 until August 31^st^ 2013) among which 1130 (41.2%), and 1594 (58.5%) were sampled from outpatients and inpatients, respectively. Of these 2724 samples, 643 (23.6%) yielded significant growth of a single organism. Among positive samples (n = 643), 35.0%, and 65.0% were isolated from outpatients and inpatients respectively. The mean age of the study population was 27.2 years, with a range of 0–75 years. Children between 0 and 17 years represented 20.6% of all patients. The female to male ratio was 1.73.

*Escherichia coli* was the most frequent uropathogen isolated (376 out of 643; 58.5%) at the Bukavu General Hospital both in outpatients and in inpatients. *Klebsiella spp.* and *Enterobacter spp.* represented 21.9% and 16.2% of uropathogens. Rare uropathogens included *Citrobacter spp.*, *Proteus spp.,* and *Acinetobacter spp*. A summary of antimicrobial susceptibility patterns of the most frequent uropathogens is presented in Table 1.

**Table 1:** Antibiotic-resistance global rate (%) of UTI isolates (n = 643) collected at the Bukavu Hospital, South Kivu, DRC

**Antimicrobial drug**	***Escherichia coli***	***Klebsiella spp.***	***Enterobacter spp.***	***Citrobacter spp.***	***Proteus spp.***	***Acinetobacter spp.***
	**(n = 376)**	**(n = 146)**	**(n = 104)**	(n = 18)*	(n = 2)*	(n = 2)*
Amikacin	9.3	2.7	2.9	5.6	100	0
Amoxycillin	16.0	13.7	17.3	16.7	100	100
Ampicillin	16.0	13.7	17.3	16.7	100	100
Amoxicillin/clavulanic acid	16.0	13.7	15.4	11.7	100	0
Ceftazidime	16.0	13.7	17.3	16.7	100	100
Ceftriaxone	15.7	13.7	17.3	16.7	100	100
Cefuroxine	15.7	13.7	17.3	16.7	100	100
Ciprofloxacine	15.4	11.7	14.4	16.7	100	100
Imipenem	0	0	1	0	0	0
Nitrofurantoin	3.5	2.1	4.8	0	0	0
Sulfamethoxazole/Trimethoprim	15.4	13.7	17.3	16.7	100	100

*Caution regarding antibiotic-resistance rates, given the low number of isolates assayed.

16.3% isolates (n = 643) displayed a MDR phenotype. Multidrug resistance rate was higher in inpatients isolates (22.1%) compared to outpatients (8.4%) (p-value <0.001). Among these MDR isolates, 92.4% displayed an ESBL phenotype. Nitrofurantoin susceptibility for *E. coli, Enterobacter spp.* and *Klebsiella spp.* was 69.6%, 78.9%, and 83.3%, respectively. Uropathogens susceptibility rates to amikacin were 42.1%, 69.6% and 77.8% for *E. coli, Enterobacter spp.* and *Klebsiella spp.,* respectively. Regarding imipenem, all isolates but one, were susceptible *in vitro*. This single imipenem non-susceptible isolate was identified as an *Enterobacter spp*. Preliminary molecular data obtained on 25 isolates showed that the CTX-M group 1 gene was the most common in ESBL-producing isolates assayed. Regarding beta-lactamases AmpC, no isolate out of the 20 isolates assayed displayed this phenotype.

## Discussion

The steady increase of bacteria resistance to antibiotics is a cause of global concern. Infections caused by resistant microorganisms often fail to respond to the standard treatment, resulting in prolonged illness and greater risk of death [30]. Available studies from several Sub-Saharian countries in Africa have highlighted unexpected high levels of resistance of uropathogens to common antibiotics [9-12,14,16,17,31]. Unfortunately, there are no data on rates of antibiotic resistance of uropathogens in DRC. In that respect, recent data on *Salmonella* isolates from blood cultures in DRC have shown high levels of antibiotic resistance [32-34].

Accordingly, the goal of this work was to provide a benchmark for the prevalence and patterns of antibiotic resistance of bacterial pathogens involved in UTIs at the Bukavu General Hospital. This study confirmed that *E. coli* was the most common bacterial uropathogen isolated from both in inpatients and outpatients at the Bukavu Hospital. Besides *E.coli*, other bacterial species such as *Klebsiella spp.* and *Enterobacter spp.* were also frequently encountered. Noteworthy, we were not able to isolate any Group B *Streptococcus (GBS)*, though GBS have been reported as causative agents of UTIs [35]. This possible bias might be linked to the sole use of CLED medium for uropathogens isolation. It has indeed been previously reported that the identification of *Streptococcus agalactiae* on CLED agar could be challenging [36].

Limitations also include the possibility of selection bias due to the fact that Bukavu Hospital mostly deals with patients who had been treated with antibiotics prior to their visit. Finally, our study may have underestimated the prevalence of UTIs by using our current threshold of 10^5^ CFU/mL of urine sample as a pre-requisite for urine culture. Several studies have shown indeed that using lower CFU threshold values improve the identification of UTIs [37,38].

Our study found that the overall prevalence of antibacterial drug resistance was lower than recently reported in neighboring countries [12,20]. A recent study in neighboring Rwanda reported resistance rates of 59.2%, 32.1% and 41.3% for amoxicillin/clavulanic acid, ceftriaxone and ciprofloxacillin, respectively [20].

Regarding the pattern of resistance in the 105 drug-resistant isolates, there was a striking low susceptibility to ampicillin, amoxicillin, amoxicillin/clavulanic, cefuroxime, ceftazidime, ceftriaxone, ciprofloxacin and sulfamethoxazole/trimethoprim (see Table 2). Conversely, susceptibility rates for rare pathogens as reported in this study (*Citrobacter spp., Proteus spp.and Acinetobacter spp.*) should be interpreted with caution, as they were based on a restricted number of isolates, and could therefore result in observational bias with overestimation of effect size. Interestingly, this study showed that ESBL-production was the main mechanism of resistance both in outpatients and inpatients at the Bukavu hospital. Global susceptibility rate of uropathogens to nitrofurantoin was 78.6%. The susceptibility of antibiotic-resistant isolates to amikacin, nitrofurantoin and to imipenem was high, which is a significant observation in terms of management of UTIs. To the best of our knowledge, this is the first study in DRC (South-Kivu Province) on the rate of resistant and/or ESBL-producing uropathogens.

**Table 2:** Antibiotic resistance rates among MDR uropathogen isolates (n = 105) at the Bukavu General Hospital

**Antimicrobial agent**	***E. coli***	***Enterobacter spp.***	***Klebsiella spp.***	Citrobacter spp.*	Proteus spp*	Acinetobacter spp*
	**(n = 57)**	**(n = 23)**	**(n = 18)**	**(n = 3)**	**(n = 2)**	**(n = 2)**
ESBL-producing (%) phenotype	98.2	100.0	100.0	100.0	100.0	0
Amikacin (%)	57.9	30.4	22.2	33.3	100.0	0
Amoxycillin (%)	100.0	100.0	100.0	100.0	100.0	100.0
Ampicillin (%)	100.0	100.0	100.0	100.0	100.0	100.0
Amoxicillin/clavulanic acid (%)	84.2	87.0	100.0	66.7	100.0	0
Ceftazidime (%)	100.0	100.0	100.0	100.0	100.0	100.0
Ceftriaxone (%)	98.2	100.0	100.0	100.0	100.0	100.0
Cefuroxine (%)	98.2	100.0	100.0	100.0	100.0	100.0
Ciprofloxacine (%)	96.5	82.6	83.3	100.0	100.0	100.0
Imipenem (%)	0	4.3	0	0	0	0
Nitrofurantoin (%)	21.1	30.4	16.7	0	0	0
Sulfamethoxazole/trimethopim (%)	96.5	100.0	100.0	100.0	100.0	100.0

*Caution regarding antibiotic-resistance rates, given the low number of isolates assayed.

Carbapenems have been recently introduced in the province for treatment of ESBL-producing *Enterobacteria*, and this study demonstrated very high susceptibility among uropathogens. Based on our findings, we advocate prescription of nitrofurantoin as the first-line antibiotics for UTIs treatment in South Kivu. Despite high susceptibility to imipenem, it is critical to warn physicians about the danger of prescribing this compound as first-line antibiotic for UTIs treatment, because this habit will likely enhance the emergence of imipenem-resistant bacteria. Likewise, several other antibiotics previously reported as active against uropathogens in other studies [7,39-41] but not currently prescribed in South Kivu should be tested against MDR-resistant uropathogens. These include drugs such as Fosfomycin [42], Piperacillin/Tazobactam [39], Fosmomycin/tromethamol [40], cefepime, tigecycline, temocillin [7,41] and other carbapemens [19]. But most importantly, it will be important to put emphasis on public and professional education towards a rational use of antibiotics, i.e. antibiotherapy based on susceptibility patterns of pathogens, promotion and evaluation of medical and veterinary practice guidelines. Curbing the spread of antibiotic resistant pathogens in the province and in the country will also entail to deal with other critical issues which should be investigated. These include controlling the influx of counterfeit drugs, and dealing with issues related to affordability of healthcare in the province.

## Conclusions

High rates of ESBL-producing Gram-negative bacteria were found among inpatients and outpatients at the Bukavu Hospital in DR Congo. Most of these ESBL-producing isolates were also multidrug resistant, except for amikacin, nitrofurantoin and imipenem for which susceptibility was high.

## Competing interests

The authors declare that they have no competing interests.

## Authors’ contributions

LMI, LK, OV, RBC and JLG participated in the design of the study. LMI oversaw the whole collection of data. LMI and LK were responsible for the laboratory assays. LMI drafted the first manuscript and all co-authors participated in the manuscript revision. All authors read and approved the final manuscript.
